# Novel Therapeutic Strategies for Metastatic Prostate Cancer Care

**DOI:** 10.1016/j.eururo.2025.06.013

**Published:** 2025-07-19

**Authors:** Alessia Cimadamore, Cristina Boixareu, Adam Sharp, Himisha Beltran, Johann S. de Bono

**Affiliations:** a Institute of Pathological Anatomy, Department of Medicine, University of Udine, Udine, Italy; b The Royal Marsden Hospital, London, UK; c The Institute of Cancer Research, London, UK; d Dana-Farber Cancer Institute, Boston, MA, USA

**Keywords:** Prostate cancer, Molecular target, Target therapy, Antibody-drug conjugates, Poly(ADP)-ribose polymerase inhibitors, Androgen therapy

## Abstract

**Background and objective:**

The elucidation of prostate cancer biology and genomics has led to new therapies improving disease outcomes with novel androgen receptor (AR) pathway inhibitors (ARPIs), taxanes, and targeted therapeutics that require disease molecular stratification.

**Methods:**

We are presenting a narrative and qualitative synthesis based on a systematic search. Medline (PubMed) and Embase (OvidSP) were searched (October 1, 2024, covering 2019–2024) using keywords and Medical Subject Headings terms; ClinicalTrials.gov and ASCO/ESMO abstracts were also reviewed. The inclusion criteria were phase 1–3 studies on molecular targets or therapies for metastatic prostate cancer with posted results. The exclusion criteria included non-English articles, reviews, meta-analyses, commentaries, case reports, duplicates, nonhuman/preclinical studies, protocols, and studies lacking molecular targets.

**Key findings and limitations:**

Targeted therapies have emerged for specific molecular subtypes of advanced prostate cancer. For instance, poly(ADP)-ribose polymerase inhibitors target DNA repair defective prostate cancer (especially *BRCA2* and *PALB2* biallelic loss). Immune checkpoint inhibitors against PD-1/PD-L1 are effective in hypermutated prostate cancer cases, especially those with mismatch repair defective (MMRd) disease. Additionally, 177Lu-PSMA-617 impacts prostate-specific membrane antigen (folate hydrolase) expressing disease. Several other major therapeutic advances are envisioned in the near future, including targeting novel cell surface proteins with T-cell engager antibody constructs, immunoconjugates, and radiopharmaceuticals. Other rational therapeutic strategies are being pursued, targeting continued AR signalling; AR cofactors, for example, P300; PI3K/AKT signalling; the PRC2 complex protein including EZH2; as well as novel synthetic lethal strategies.

**Conclusions and clinical implications:**

A rapidly evolving standard of care is anticipated for metastatic prostate cancer, making it imperative that rational registration trial designs incorporating multipurpose biomarkers to accelerate anticancer drug development are pursued.

## 1. Introduction

Despite clinically important advances, prostate cancer (PCa) remains a leading cause of cancer death for men worldwide [1]. As a hormone-driven disease, upfront systemic treatment for metastatic PCa relies on androgen deprivation therapy (ADT) and androgen receptor (AR) pathway inhibitor (ARPI) therapy, with docetaxel added increasingly [2]. However, castration resistance develops over time [3]. Several drugs are approved for metastatic castration-resistant PCa (mCRPC), including ARPIs, taxanes targeting tubulin, and DNA damage inducing radioligand therapies (eg, radium-223 and 177Lu-PSMA-617) and poly(ADP)-ribose polymerase (PARP) inhibitors (PARPis), the latter imparting a major benefit for patients with homologous recombination repair defective (HRD) tumours. Despite these advances, the median overall survival for men with mCRPC remains <5 yr [4], highlighting an urgent need for new therapies. Growing insights into mCRPC biology are driving the development of biomarker-driven therapeutics [2,5]. This review outlines emerging strategies for treating advanced PCa.

## 2. Methods

We are presenting a narrative and qualitative synthesis based on a systematic search. Medline (PubMed) and Embase (OvidSP) were searched (October 1, 2024, covering 2019–2024) using keywords and Medical Subject Headings terms listed in the Supplementary material. ClinicalTrials.gov and ASCO/ESMO abstracts were also reviewed.

Phase 1–3 studies on molecular targets or therapies for metastatic PCa with posted results were included. Non-English articles, reviews, meta-analyses, commentaries, case reports, duplicates, nonhuman/preclinical studies, protocols, and studies lacking molecular targets were excluded.

**Fig. 1 –:**
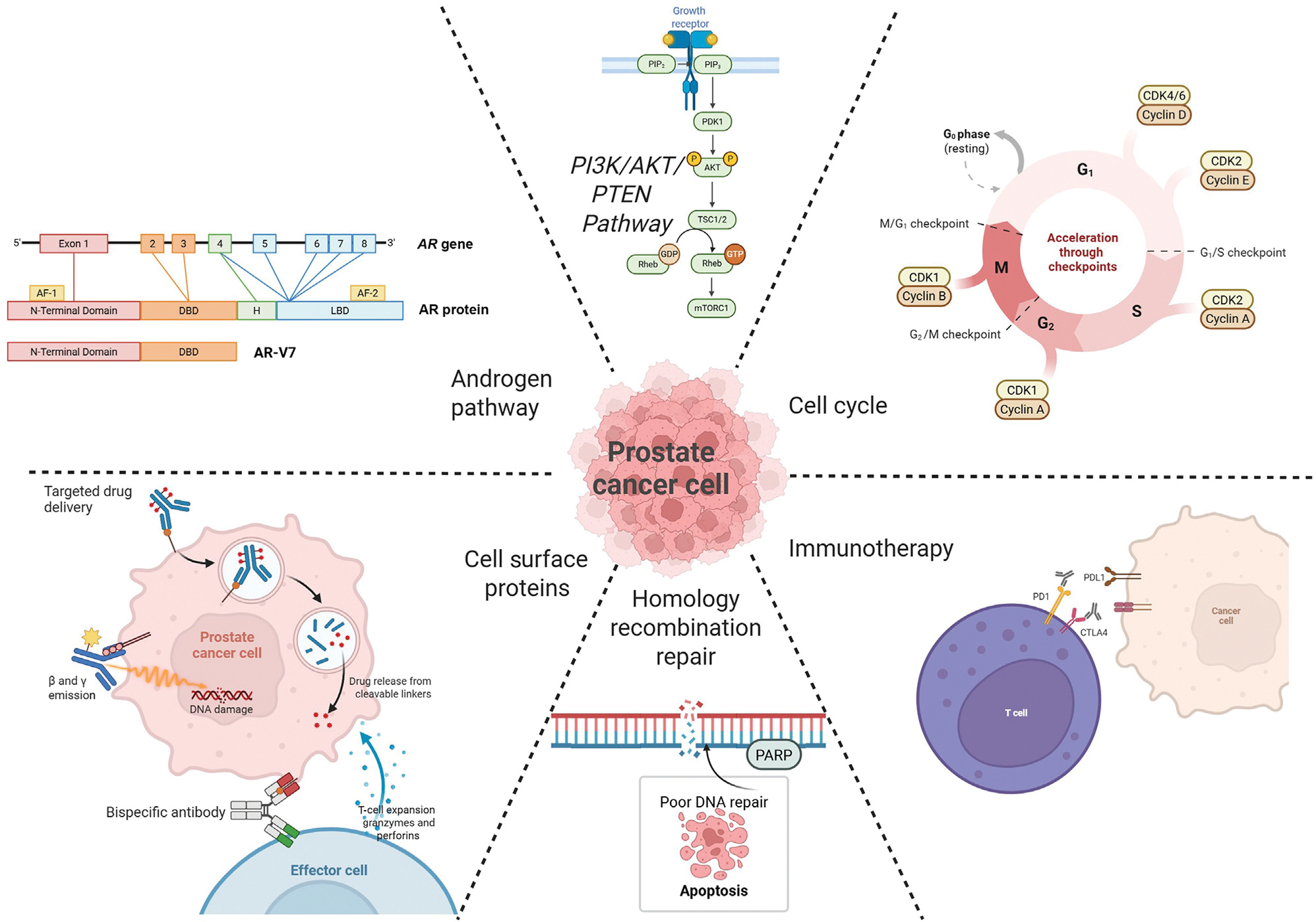
Molecular target pathways. AR = androgen receptor; AR-V = AR variant; PARP = poly(ADP)-ribose polymerase.

**Fig. 2 –:**
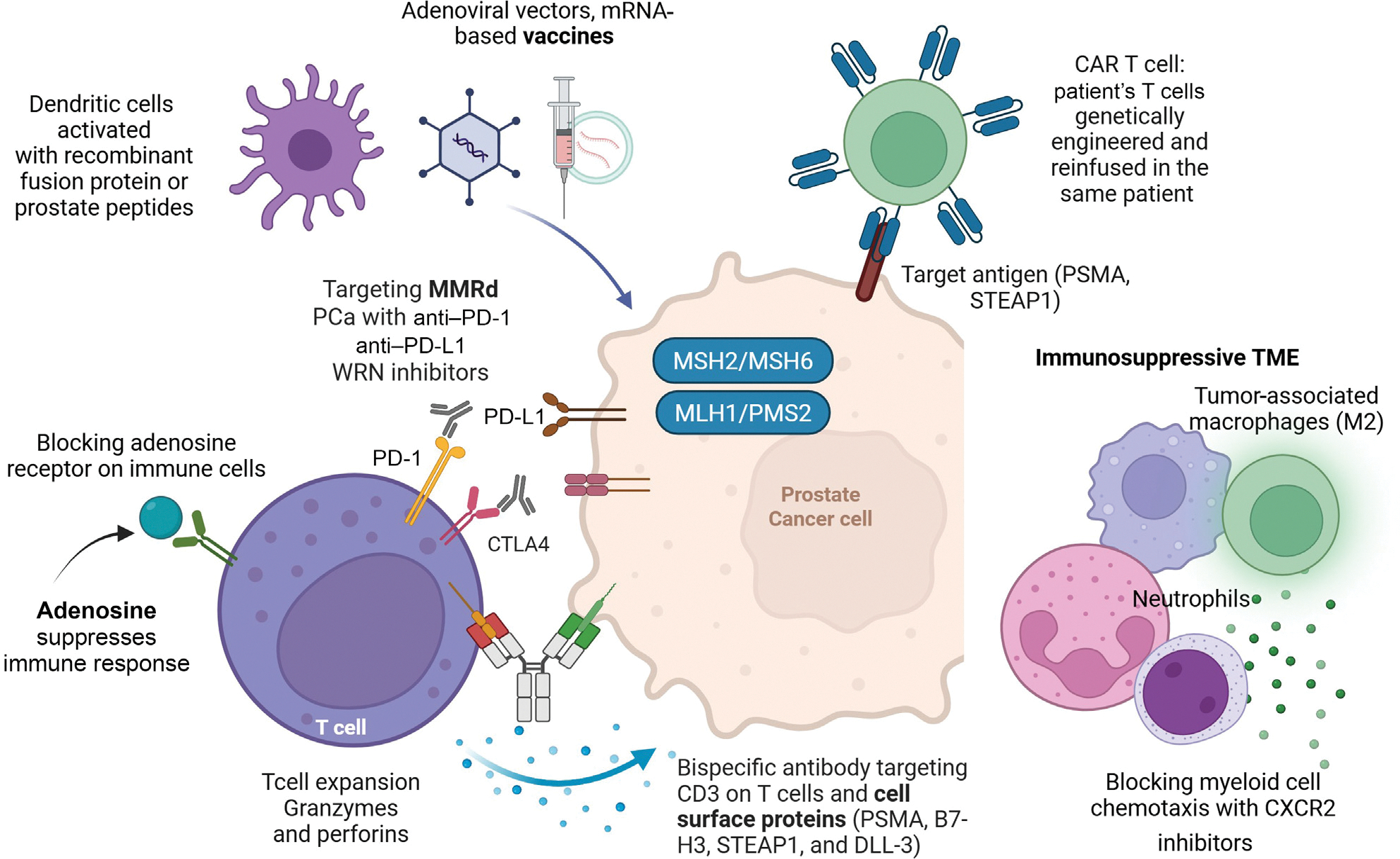
Immunotherapy strategies to target advanced prostate cancer. MMRd = mismatch repair defective; PCa = prostate cancer; PSMA = prostate-specific membrane antigen; STEAP1 = six-transmembrane epithelial antigen of prostate 1; TME = tumour microenvironment.

After deduplication, three authors (A.C., J.B., and C.B.) independently screened the studies (see Supplementary Fig. 1). The key molecular pathways appear in Figs. 1 and 2.

## 3. Results

### 3.1. Targeting continued AR pathway signalling

The AR pathway remains central to PCa biology, supporting cell survival, proliferation, and resistance. While ADT and ARPIs improve survival for metastatic PCa, resistance develops through divergent mechanisms typically converging on increased AR signalling, including increased ligand biosynthesis, *AR* amplification, *AR* splicing, and *AR* activating mutations, as well as AR-independent mechanisms. Targeting persistent AR signalling remains a priority; cross-resistance has limited sequencing of currently available ligand-binding domain (LBD) targeted ARPIs due to limited antitumour activity [6]. Targeting constitutively active AR splice variants (AR-SVs) that lack the LBD remains an unmet need despite multiple unsuccessful efforts, including niclosamide (putative AR-V7 inhibitor), TAS3681 (targeting AR and AR-SV generation), EPI-7386 (putative AR N-terminal inhibitor), galeterone (AR degrader and CYP17 inhibitor), and ONCT-534 (putative AR N-terminal and LBD inhibitors) [7–11]. Future strategies will require superior compounds, predictive biomarkers detecting AR-SVs such as circulating tumour cell (CTC) protein expression, and arguably rational combinations targeting coexisting resistance pathways such as PI3K/Akt/mTOR, epigenetic alterations, and disease heterogeneity.

Strategies targeting AR degradation via E3 ubiquitin ligases are also being pursued but primarily target the AR-binding domain. Bavdegalutamide degrades wild-type and mutant ARs with antitumour activity against AR-mutated mCRPC (50% decline in prostate-specific antigen [PSA] value [PSA50]: 46%, 30% decline in PSA value: 58%); this is ineffective against AR-SVs lacking the LBD [12]. A phase 1/2 trial of the AR degrader ARV-766, with activity against certain AR mutations where bavdegalutamide is ineffective, is underway (NCT05067140). BMS-986365, another AR degrader and antagonist, also demonstrated PSA responses with 6.3-mo radiographic progression-free survival (rPFS; 50% PSA50 and 8.3 mo rPFS at 900 mg BID) in a phase 1 trial in mCRPC after ARPI and taxane therapy [13]. Adverse events (AEs) included QTc prolongation (47%, 9% grade 3) managed by dose reduction. A phase 3 trial in mCRPC is comparing BMS-986365 with a second ARPI or docetaxel [14]. Preliminary studies suggest that AR-mutated PCa cases have low AR-SV expression, possibly explaining this AR degrader antitumour activity.

An alternative strategy to blocking continued AR signalling is CYP11 inhibition, upstream of CYP17; opeveostat (CYP11A1 inhibitor) was evaluated in the phase 1/2 CYPIDES trial for heavily pretreated mCRPC. Dexamethasone and fludrocortisone supplementation were required to prevent adrenal insufficiency; grade ≥3 AEs (67%) included fatigue, anaemia, muscle spasms, insomnia, diarrhoea, hyponatraemia, and hyperkalaemia. PSA50 responses were 53.0% in AR-LBD mutation-positive and 14.7% in mutation-negative patients, with an objective response rate (ORR) of 18.6% in AR-LBD mutated cases [15]. These findings may stem from lower-affinity steroid hormones activating mutated *AR* [16]. A phase 3 trial is comparing opevesostat with ARPI switch in post-ARPI post-taxane mCRPC cases (NCT06136624).

Although corticosteroids block adrenal testosterone synthesis, these can also drive AR mutations, including L702H [17], which promotes ARPI resistance. Glucocorticoid receptors (GRs) and ARs, both nuclear hormone receptors, have overlapping cistromes due to many shared DNA response elements [18], allowing GR signalling to compensate for AR inhibition and impact ARPI sensitivity [18]. In the AFFIRM enzalutamide trial, corticosteroid use correlated with reduced overall survival (OS) and rPFS [19]. Patients with low GR/AR ratios had better PSA progression-free survival (PFS; 46% vs 22.4%), rPFS (28.9% vs 10%), and OS (75.2% vs 48%) than those with high ratios [18]. However, the GR antagonist relacorilant with enzalutamide for mCRPC after enzalutamide has demonstrated limited efficacy [20]; two further phase 1 trials of GR antagonists (ORIC-101, mifepristone) with enzalutamide were terminated due to insufficient benefit and tolerability challenges [21,22]. Further studies should pursue strategies selectively targeting GRs in tumour cells and consider selecting patients whose tumours express high GRs but lack AR-SVs.

Microbiota may contribute to ARPI resistance by converting androgen precursors into androgenic metabolites [23]; a phase 1 trial (NCT06126731) is studying microbiome sculpting to counter this.

AR signalling can also be disrupted by targeting transcriptional coactivators and chromatin regulators, including BET BRD2/3/4, P300 histone acetyltransferase [24], and histone deacetylase (HDAC), impacting PCa genomic and epigenetic aberrations [25,26]. HDAC inhibitors have demonstrated preclinical mCRPC antitumour activity and synergy with ARPIs, prompting clinical evaluation [27].

BET and P300 inhibition can block AR and AR-SV signalling. BET inhibitors, including molibresib, birabresib, and ZEN-3694, had limited antitumour activity, narrow therapeutic windows, and haematological toxicity in clinical trials, restricting their development [28–31]. P300 blockade helps reverse ARPI resistance, and selective P300 degraders are under evaluation [32].

Another novel strategy leveraging high AR expression now undergoing clinical evaluation is RIPTACs, which recruit ARs and an essential cellular protein into a stable ternary complex, inhibiting the essential protein’s function and leading to cell death in AR-positive PCa cells [33].

### 3.2. Targeting PI3K/AKT/PTEN signalling

The PI3K/AKT/PTEN pathway, frequently activated in PCa due to PTEN loss, correlates with poor prognosis. Targeting p110α/β and AKT remains under clinical investigation [34,35]. Crosstalk between the PI3K/AKT/mTOR and AR pathways exists, where AR inhibition increases AKT signalling, making this pathway a key therapeutic target [31]. In the phase 3 IPATential150 trial, ipatasertib (AKT inhibitor) plus abiraterone had a minimal rPFS benefit (2 mo) in mCRPC with PI3K/AKT activating aberrations with no OS benefit [36–38]. Furthermore, a recent press release has reported that the phase 3 trial of docetaxel and the AKT inhibitor capivasertib for post-ARPI mCRPC was discontinued early since it was deemed unlikely to meet its primary endpoint [39]. A phase 3 trial is evaluating capivasertib with abiraterone in PTEN-deficient metastatic hormone-sensitive PCa (mHSPC; NCT04493853) [40], with a recent press release reporting that this trial has met its primary endpoint of improved rPFS compared with placebo/abiraterone [41]. Optimising biomarkers, such as ctDNA profiling for PI3K/AKT pathway mutations, and understanding the impact of co-occurring molecular alterations may help improve future patient selection. Combination strategies, including AKT inhibitors with DNA damage response agents, MCL-1 inhibition, or immunotherapy, are being explored as PTEN loss promotes resistance to these therapies [42,43].

### 3.3. CDK4/6 inhibition

Androgens promote PCa growth at least in part by upregulating cyclin D, which complexes with CDK4/6 to phosphorylate Rb, releasing E2F and driving cell cycle progression; CDK4/6 also enhances AR transcriptional activity. CDK4/6 inhibitors are approved for HR+, human epidermal growth factor receptor 2 (HER2)− breast cancer [40,41,44]. *RB1*-deficient or cyclin-E–overexpressing tumours do not respond to these therapies [45]. A phase 2 trial (NCT02059213) of ADT ± palbociclib in *RB1*-intact de novo mHSPC failed to improve PSA response or PFS. A phase 1/2 trial of docetaxel, prednisone, and ribociclib (reduced to 400 mg daily) had median rPFS of 8.1 mo in mCRPC patients; neutropenia (37%) was the most common grade ≥3 AE. Abemaciclib, tested for heavily pretreated mCRPC unselected for *RB1* status, did not meet its ORR primary endpoint. CYCLONE 2/3 trials of abemaciclib with abiraterone/prednisone for first-line mCRPC and high-risk mHSPC showed no survival benefit, but did not pursue *RB1* selection [46]. Further work should explore potential signals of efficacy in molecularly defined subgroups to ensure that promising avenues are not dismissed prematurely.

### 3.4. Targeting non-AR driven mCRPC

Loss of *TP53* and *RB1* is associated with AR independence and lineage plasticity in mCRPC [47], often identified in treatment-emergent neuroendocrine PCa (NEPC) [48]. In metastatic PCa, biallelic inactivation of *TP53* (mutation or copy loss) or *RB1* (mainly copy loss) occurs in ~40–50% and ~12% of cases, respectively, and both genes are lost in ~4% of cases [47]. Rb regulates G1-to-S phase progression via CDK4/6, and its loss can confer resistance to endocrine, targeted, and CDK4/6 therapies, partly through the E2F/EZH2/NSD2 axis. This has led to strategies targeting replication stress, including cytotoxic agents such as platinum and nucleoside analogues; topoisomerase, ATR, or CDC7 inhibitors; as well as Aurora-A kinase (AURKA). Targeting epigenetic changes associated with lineage plasticity have supported development of drugs targeting EZH1/2, and more recently NSD2 [49,50].

#### 3.4.1. Aurora kinase inhibitors

NEPC shares histological and molecular features with small cell lung cancer, has poor prognosis [51], and is characterised by AURKA and N-Myc overexpression with lower AR signalling, decreasing PSA and prostate-specific membrane antigen (PSMA) levels [52]. Alisertib (AURKA inhibitor) showed modest efficacy in a metastatic NEPC phase 2 trial, with a 6-mo rPFS rate of 13.4% and median OS of 9.5 mo [53]. Biomarker selection such as RB1 loss or AURKA amplification was not used in this study.

#### 3.4.2. CDC7

CDC7, a kinase essential for DNA replication and repair under replication stress, is reported to be a dependency in NEPC [54]. A CDC7 inhibitor, simurosertib, is in clinical trials (NCT02699749 and NCT03261947) with neutropenia as the main toxicity [55]. Other replication stress-targeting strategies include DNA repair gene inhibition, including Wee1 and ATR kinases [56]. Predictive biomarkers are urgently needed for these efforts.

#### 3.4.3. EZH2

Increased E2F signalling drives EZH2 (arguably EZH1) function; this impacts biological changes including chromatin modifications. EZH2 can drive AR signalling through non-canonical functions and modulate lineage plasticity, with EZH2 inhibition resensitising to ARPIs [49]. Trials evaluating EZH2 inhibitors, including PF-06821497, with ARPIs are being pursued [57,58], and phase 3 evaluation is underway (NCT06551324 and NCT06629779). Mevrometostat, a selective EZH2 inhibitor, in a phase 1 after abiraterone and chemotherapy study in mCRPC, in combination with enzalutamide, was associated with a 49% relative reduction in the rate of progression or death, corresponding to an 8-mo improvement in median rPFS. The combination was tolerable (53.7% grade ≥3 AEs vs 42.5% with enzalutamide alone) [59], and randomised phase 2 data have been presented but not published.

### 3.5. Targeting DNA repair defects and PARPis

PARPs are key to DNA repair; HRD tumours rely on PARP activity, making them vulnerable to PARPis. Loss of *BRCA1/2*, *PALB2*, and other HR genes sensitises to these agents, which are now the standard of care for HRD mCRPC [60,61]. The PROPEL, MAGNITUDE, and TALAPRO-2 phase 3 trials demonstrated a significant rPFS benefit from PARPis and ARPIs in mCRPC patients, with maximal efficacy against tumours with *BRCA2* biallelic loss and limited efficacy against tumours without HRD [62–64]. Multiple trials are testing PARPis alone or in combination. Concerns remain about longer-term use in earlier-stage disease with DNA damage in haemopoietic progenitors resulting in secondary haematological malignancies [65]. Efforts to develop PARP1 selective inhibitors have shown limited evidence for this, causing decreased haematological toxicity. Combination strategies, including the pursuit of sensitisation to DNA damaging agents, include topoisomerase inhibitors carrying immunoconjugates and radionuclides, and rational combinations with PolQ inhibitors to prevent the emergence of BRCA reversion mutations that cause resistance in BRCA mutation carriers.

### 3.6. Targeting cell surface proteins

#### 3.6.1. PSMA-targeted radioligands

**Fig. 3 –:**
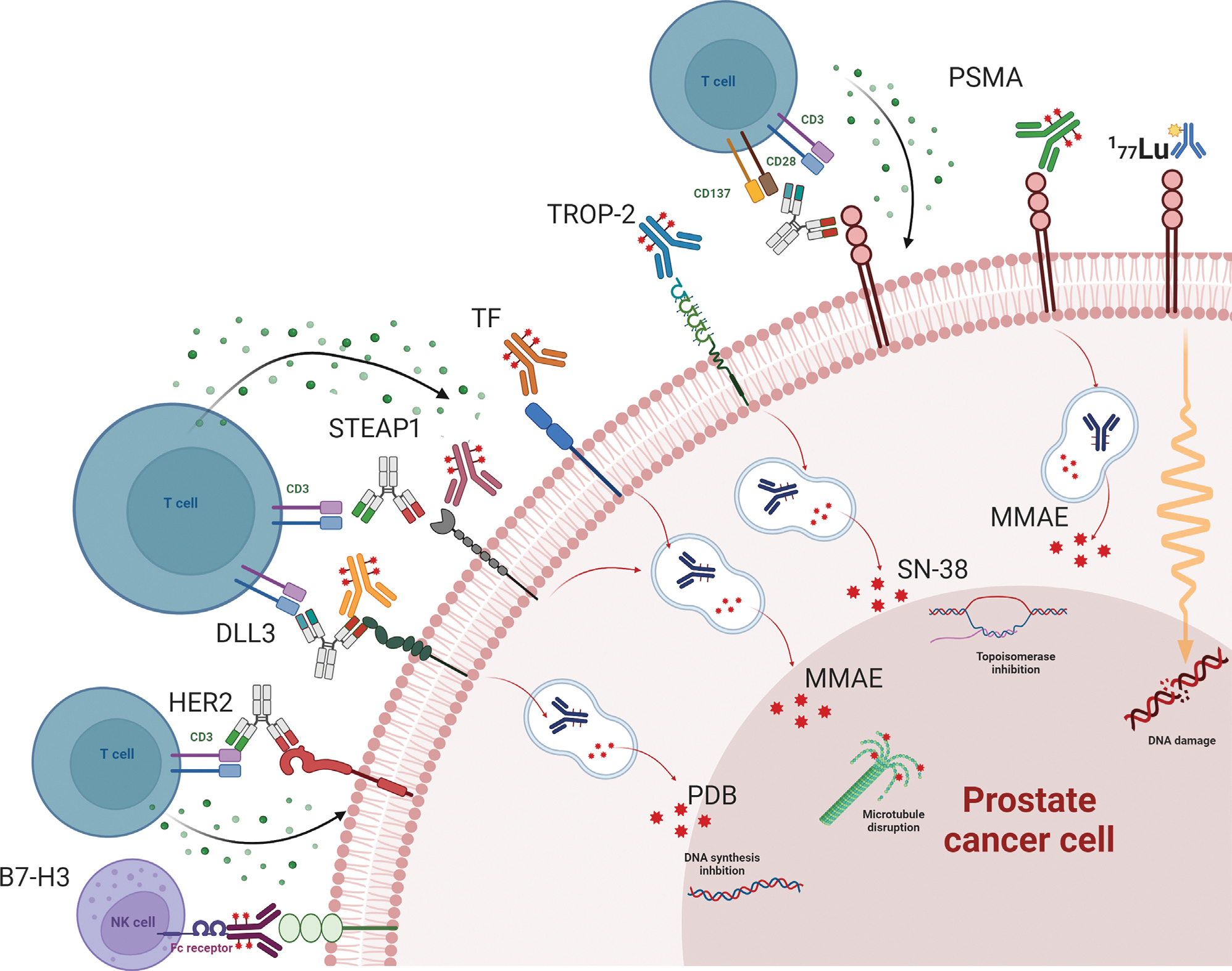
Targeting cell surface proteins. The figure shows surface target proteins currently under investigation in prostate cancer. The different surface proteins can act as targets for radioligand therapies (ie, PSMA), for ADC therapies, delivering different payloads with different mechanisms of actions or as targets of bispecific antibodies against CD3, CD28, and CD137 on T-cells to stimulate an antitumour response through T-cell expansion and secretion of cytokines, granzymes, and perforins. ADC = antibody-drug conjugate; HER2 = human epidermal growth factor receptor 2; MMAE = monomethyl auristatin E; PSMA = prostate-specific membrane antigen; STEAP1 = six-transmembrane epithelial antigen of prostate 1; TF = tissue factor.

PSMA is a validated theranostic target using alpha- or beta-emitting isotopes (Fig. 3). Alpha-particles deposit energy within 50–100 μm, inducing lethal DNA double-strand breaks, while beta-particles have longer ranges (1–5 mm), impacting larger tissue volumes [66]. The beta-emitter 177Lu-PSMA-617 is clinically approved, initially based on the VISION trial, comparing it with the standard care therapy (including a second ARPI, bisphosphonates, radiation therapy, denosumab, or glucocorticoids), although the TheraP trial did not show OS superiority over cabazitaxel. Recently, 177Lu-PSMA-617 was approved by the U.S. Food and Drug Administration in the predocetaxel mCRPC setting based on the PSMAfore phase 3 trial showing improved rPFS, ORR, and PSA50 response but no OS benefit, probably due to high cross-over from the ARPI arm [67]. The ENZA-P trial reported longer PSA PFS with enzalutamide + 177Lu-PSMA-617 versus enzalutamide alone (13 vs 7.8 mo) in docetaxel- and ARPI-naïve mCRPC patients [68]. The on-going trials include LuPARP (177Lu-PSMA-617 + olaparib, NCT03874884), PSMAddition (177Lu-PSMA-617 + ARPI/ADT, NCT04720157), and PRINCE (177Lu-PSMA-617 + pembrolizumab, NCT03658447).

The alpha-emitter 225Ac-PSMA-617 showed 57% PSA50 in pretreated mCRPC patients, though dry mouth occurred in 95% of patients after five cycles [69]. In trials involving >150 beta-emitter–resistant patients with mCRPC, 225Ac-PSMA-617 was associated with >65% PSA50 responses with limited nephrological and haematological toxicities [70,71].

Other radioligand targets currently in phase 1 NEPC trials include gastrin-releasing peptide receptor and somatostatin receptor-2 (NCT06379217).

#### 3.6.2. Antibody-drug conjugates in PCa

Antibody-drug conjugates (ADCs) combine a monoclonal antibody targeting specific tumour antigens with a cytotoxic payload, enabling targeted therapy while limiting exposure to healthy tissues. Clinical trials are underway targeting cell surface proteins including PSMA, TROP-2, six-transmembrane epithelial antigen of prostate 1 (STEAP1), tissue factor (TF), DLL-3, HER2/3, B7-H3, and Nectin-4.

##### 3.6.2.1. PSMA.

PSMA-targeted ADCs have shown limited responses to date. In a phase 2 trial of a PSMA ADC linked to monomethyl auristatin E (MMAE), 14% PSA50 responses were observed in mCRPC patients, with better results in chemotherapy-naïve patients [72,73]. Another PSMA ADC with DM1 payload showed an 8% PSA50, hampered by neurotoxicity due to linker instability [74].

##### 3.6.2.2. Trophoblast cell surface antigen-2.

Sacituzumab govitecan, a Trop-2-directed ADC, was associated with one complete response in a phase 1/2 trial (NCT01631552). Trials are on-going for sacituzumab govitecan in mCRPC patients after ARPI (NCT03725761) and in combination with adenosine receptor antagonists (NCT04381832) [75].

##### 3.6.2.3. Tissue factor.

Tisotumab vedotin, targeting TF, showed an ORR of 15.6% in a phase 1/2 trial (NCT02001623) across cancer types, including PCa; antitumour activity with this tubulin-binding drug payload in mCRPC cases was modest, but since TF is highly expressed in mCRPC, it remains a target of interest, warranting further investigation in this setting [76].

##### 3.6.2.4. Six-transmembrane epithelial antigen of prostate 1.

Six-transmembrane epithelial antigen of prostate 1 (STEAP1)-targeted ADCs such as DSTP3086S reported 18% PSA50 in a phase 1 trial but were discontinued due to safety concerns [77]. A STEAP1-CD3 bispecific showed a 49% PSA50 and 24% ORR, with manageable grade 1/2 cytokine release syndrome (CRS) in 72% of patients in phase 1 trials. Further trials will test combinations with abiraterone or enzalutamide (NCT04221542) [78].

##### 3.6.2.5. Human epidermal growth factor receptor 2 and 3.

HER2 protein is expressed, largely at low levels, in mCRPC patients, although some patients have tumours with higher expression that can respond to HER2 targeting agents (NCT05057013). HER3 protein overexpression is common in mCRPC, associated with poor outcomes, and can be targeted by HER3 targeting agents such as immunoconjugates or radioimmunoconjugates [79]. A phase 1 trial combining anti-HER3 mAb with enzalutamide is on-going in PTEN-preserved mCRPC with HER3 expression (NCT05057013).

##### 3.6.2.6. B7-H3.

B7-H3, part of the B7 superfamily that includes B7-H1(PD-L1), is highly expressed in most mCRPC cases, with its expression regulated in part by AR signalling. A naked B7-H3 targeting antibody, enoblituzumab, had modest single-agent antitumour activity with no unexpected safety issues (NCT02923180) [80]. B7-H3 ADCs such as I-DXd (now MK-2400) and vobra-duo have clinically significant antitumour activity, although payload toxicity concerns remain (NCT03729596 and NCT06242470) [81]. B7-H3 targeting ADCs are now moving into phase 3 clinical trial evaluation.

##### 3.6.2.7. Nectin-4.

Enfortumab vedotin, a Nectin-4–directed MMAE-conjugated ADC, has shown an OS benefit in monotherapy and in combination with pembrolizumab in bladder cancer [82,83] and is being investigated in a phase 2 study in postdocetaxel and ARPI-treated mCRPC patients, as high membranous Nectin-4 expression was found in metastases of PCa models [84]. In stage I, seven of 11 patients had a protocol-defined response (PSA50, ORR, CTC, and/or ≥6 mo on treatment); eight discontinued due to progressive disease, and enrolment in stage 2 is on-going.

### 3.7. Immunotherapy

Immune checkpoint inhibitors (ICIs) have limited efficacy against advanced PCa. Pembrolizumab monotherapy achieved a 5% ORR in PD-L1–positive and a 3.5% ORR in PD-L1–negative patients, with median OS of 9.5 and 7.9 mo, respectively [85]. The phase 3 KEYNOTE-921 (pembrolizumab/placebo + docetaxel) failed to improve rPFS and OS, while pembrolizumab with ARPIs or PARPis also showed no benefit in unselected mCRPC patients (KEYNOTE-641, KEYNOTE-991, and KEYLYNK-010) [86,87]. Atezolizumab alone or with enzalutamide had similarly disappointing results. The CONTACT-02 trial (cabozantinib + atezolizumab vs ARPI switch) resulted in a 2-mo improvement in PFS (hazard ratio 0.65) but no OS benefit [88]. Ipilimumab + nivolumab also showed low ORR and PSA responses [89]. Radium-223 is reported to enhance immunogenicity and PD-L1 expression in mCRPC patients [90], but in a phase 2 trial, the addition of pembrolizumab did not increase CD4+/CD8+ T-cell infiltration in paired bone biopsies and showed no improvement in rPFS or OS in a biomarker-unselected population [91]. Other combined radioligand therapies with ICIs are undergoing evaluation [92,93].

#### 3.7.1. Mismatch repair defective PCa

Mismatch repair deficiency (dMMR) PCa responds favourably to ICIs, with anti–PD-1 ICIs approved as tumour-agnostic monotherapy for such tumours [94,95]. The INSPIRE trial (NCT03570619) tested nivolumab and ipilimumab in biomarker-selected mCRPC population and found dMMR as the strongest response predictor, with 32.7 versus 4 mo rPFS in all patients. Other biomarkers (CDK12 inactivation, tumour mutational burden >7 mut/Mb, and BRCA2 mutations) were less predictive. ICIs have limited potential for broader PCa subgroups [96,97]. WRN inhibitors can target dMMR tumours via synthetic lethality from unresolved replication stress [98].

#### 3.7.2. Adenosine receptor

Adenosine suppresses immune responses in the tumour microenvironment by binding its receptors on immune cells. Inhibition of adenosine production (CD73/CD39) or blockage of its receptors enhances immunotherapy activity preclinically. Clinical trials have failed to demonstrate any benefit in mCRPC patients; further trials of these agents with T-cell engagers could demonstrate whether this is a valid target [99].

#### 3.7.3. T-cell engagers

Bispecific T-cell engager antibodies (bsAbs) targeting PSMA, B7-H3, STEAP1, and DLL3, combined with targeting CD3 T-cell stimulation, show promising antitumour activity [78]. PSMA-CD3 bsAbs have led to PSA responses, some long term [100]. STEAP1, a metalloproteinase rarely expressed in healthy tissues but overexpressed in mCRPC patients, was targeted in a phase 1 trial showing a 24% ORR and 48% disease stabilisation with an impressive PSA response rate [78]. DLL3, expressed in 77% of NEPC cases, is targeted by tarlatamab, a DLL3-CD3 bsAb approved for small cell lung cancer. The DLL3 T-cell engager MK-6070 shows promise in early trials (Beltran H, ESMO 2024) [101], while the DLL3 ADC Rova-T was ceased due to a lack of benefit and toxicity [102]. Challenges for T-cell engagers include the development of antidrug antibodies and CRS, mitigated through step dosing, CD3 affinity reduction, and tumour-conditional activation via cleavable masking strategies. Other approaches engage γδ T cells (NCT05369000) or NK cells (NCT06056791), associated with antitumour activity and a lower CRS risk.

Encouraging activity has been observed with PSMA-CD3 T-cell engagers, including acapatamab, JNJ-08137, and pasotuxizumab, with PSA50 responses of 30.4%, 5.1%, and 25.5%, respectively, in phase 1 trials, linking T-cell activation to PSA declines [100]. On-going trials in the mCRPC (NCT04104607) and biochemical recurrence (NCT05646550) settings continue. Costimulatory combinations of PSMA-CD28 with cemiplimab showed promise in a phase 1/2 trial [103] and PSMA-CD137 molecule (CB307) combined with pembrolizumab is awaited (NCT04839991).

#### 3.7.4. CAR T-cells

CAR T-cell therapies targeting PSMA (NCT04227275), B7-H3 (NCT04483778), and STEAP1 (NCT06236139) have potential, but have faced challenges due to tumour immunosuppression, CRS, and cost. Innovations include dual-antigen targeting, CAR-γδT-cells, and CAR-NK cells (NCT03692663), which offer HLA-independent targeting and improved safety due to their limited lifespan. Fusion CAR constructs incorporating SIRPα-Fc domains may enhance antitumour activity by reducing CAR T-cell exhaustion.

PSMA-CAR T-cell therapy led to stable disease in four of five evaluable patients with PSA declines [104]. On-going trials include PSMA-CAR-T and dual-targeting TGF-β and PSMA therapies to resist TGFβ and mitigate immunosuppression or uPAR-CART targeting senescent cells (NCT04227275 and NCT03089203) [105]. STEAP2, a prominent PCa antigen with consistent cell surface expression across all disease stages and limited in normal tissues, can be targeted using a multifaceted approach, an armoured STEAP2 CAR-T, enhanced with a dominant-negative TGF-β type II receptor to improve activity in the TGF-β–rich, immunosuppressive PCa environment (NCT06267729).

Next-generation CAR T-cells address prior drawbacks, including limited antigen sensitivity and T-cell exhaustion from persistent signalling. For instance, truncated intracellular domains of cytokine receptors and STAT3 motifs help maintain T-cell function by delaying terminal differentiation. “AND gate circuits” improve precision by requiring simultaneous detection of multiple tumour antigens, reducing off-target activation. “AND-NOT gate designs” aim to suppress CAR T-cell activation in response to normal cell antigens.

#### 3.7.5. Vaccines

PCa vaccine development has faced challenges due to antigen heterogeneity and immune evasion. Platforms including adenoviral vectors, plasmid DNA with checkpoint inhibitors (NCT02616185), tetanus-epitope targeting platforms (NCT04701021), and mRNA-based vaccines targeting STEAP1, PSA, PSCA, and PSMA or targeting kallikrein-2/3, HOXB13, and NK3 (NCT04382898) demonstrate immunogenicity but need efficacy improvement. While antigen-specific cytotoxic T-cell activation correlates with improved outcomes, T-cell exhaustion, poor tumour infiltration, epitope downregulation, and impaired antigen presentation remain major limitations.

Some argue that PCa vaccines may be more efficacious as immunomodulatory agents, potentially enhancing antigen spread and addressing tumour heterogeneity when combined with CAR T-cell therapies. Similarly, dendritic cell–based immunotherapy, despite initial promise considering the data that led to the approval of sipuleucel-T, has failed to elicit robust antitumour responses. Efforts to enhance dendritic cell–mediated immunity have focused on PSMA-HLA-A2–restricted peptides and multiepitope approaches to counter antigen loss. Lipid nanoparticle encapsulation, prolonged RNA stability, and CAR T-cell combinations hold promise for enhancing therapeutic responses.

#### 3.7.6. Targeting myeloid cell chemotaxis

The tumour microenvironment can impact response and resistance to systemic therapies. Immunosuppressive myeloid-derived suppressor cells and tumour-associated macrophages, particularly M2 polarised, secrete cytokines and growth factors that suppress T-cell activation, impair antigen presentation, and promote tumour proliferation. Neutrophils contribute to PCa progression by forming neutrophil extracellular traps and web-like chromatin structures composed of DNA, histones, and antimicrobial proteins, which obstruct drug penetration, activate oncogenic pathways such as MAPK and NF-jB, and promote cancer cell survival and metastasis. CXCR2 inhibitor blocking of myeloid cell chemotaxis has shown potential for reversing endocrine resistance in mCRPC [106].

## 4. Discussion

It is envisioned that in the near future, there will be further major changes to PCa care with several new therapeutic strategies impacting the care of advanced PCa. Novel therapies that are likely to get approved include immunotherapy strategies such as T-cell engagers, radioligand therapies, and ADCs. Concerns remain, however, on focusing many new available therapies in the mHSPC setting, without molecular stratification, since costly late trial failures will limit advances and not all the agents being tested can be approved in this space. More strategic pursuit of therapeutic combinations, utilising predictive and response biomarkers, and evaluating minimal residual disease focused on eradicating persistor (including senescent, non-cycling) cells, are now needed to raise the bar and maximise benefit from these therapies. Strategies focused on blocking lineage plasticity, by, for example, targeting chromatin modifying enzymes or reversing luminal to basal switch, to herd tumour cells away from cell states resistant to endocrine treatments need to be studied. Moreover, the paracrine interactions that appear to be so crucial to PCa survival growth need to be considered in these efforts and targeted systematically, including interactions with myelomonocytic cells. Furthermore, simple approaches focused on altering dysbiosis in PCa sufferers may also be inexpensive ways to impact outcomes. Overall, these therapies need to add years of extra life or increase cure rates to deliver cost effectiveness, which perhaps is more achievable in the adjuvant setting. Circulating biomarker-guided approaches may be key to delivering such advances and transforming PCa care.

## 5. Conclusions

Many novel therapeutic strategies for improving mCRPC care have emerged, with some transforming clinical care. There is incontrovertible evidence for the need for improved molecular stratification and rational biologically driven therapeutics. Tissue, circulating, and imaging biomarkers are increasingly recognised as having predictive and prognostic values. Ultimately, integrating biomarkers is likely to be key to managing PCa resistance. Further advances over the next decade are envisioned and likely to transform PCa care further.

## Supplementary Material

Supplementary Figure

Supplementary data to this article can be found online at https://doi.org/10.1016/j.eururo.2025.06.013.

## ADVANCING PRACTICE

### What does this study add?

This collaborative review outlines emerging strategies for treating prostate cancer by reporting the most up-to-date results from international on-going trials. It also reports potential drawbacks/limited responses or side effects that emerged during investigation, which limited further evaluations.

### Clinical Relevance

This review underscores the critical need for molecular stratification in the management of patients with metastatic prostate cancer, highlighting how precision-targeted therapies, such as PARP inhibitors for DNA repair-deficient tumours and PSMA-targeted radioligands, are reshaping therapeutic paradigms. For uro-oncologists, these advances emphasize the necessity of integrating genomic profiling into routine practice to identify actionable alterations and optimize patient selection for emerging therapies. Furthermore, the development of novel immunotherapies and antibody-drug conjugates heralds a transformative era in advanced prostate cancer care, demanding continuous clinical adaptation and biomarker-driven treatment planning. Associate Editor: Gianluca Giannarini, M.D.

### Patient Summary

Prostate cancer is still a leading cause of cancer death in men despite recent drug approval. This review aims to report and discuss the on-going lines of prostate cancer research under investigation in clinical trials. Experimental strategies include targeting the most frequently activated oncogenic pathways such as the androgen receptor and PI3K/AKT/PTEN signalling or the DNA repair machinery to induce tumour cell death. Novel approaches exploit the expression of specific surface proteins on cell membranes to deliver toxic drugs inside the tumour cell or to sensitise the immune system against the tumour itself.
