# Supplementary figures and images for: Novel Therapeutic Strategies for Metastatic Prostate Cancer Care
Source: Eur Urol. Author manuscript; Available in PMC 2026 Mar 3. (PMC12954635; doi:10.1016/j.eururo.2025.06.013)

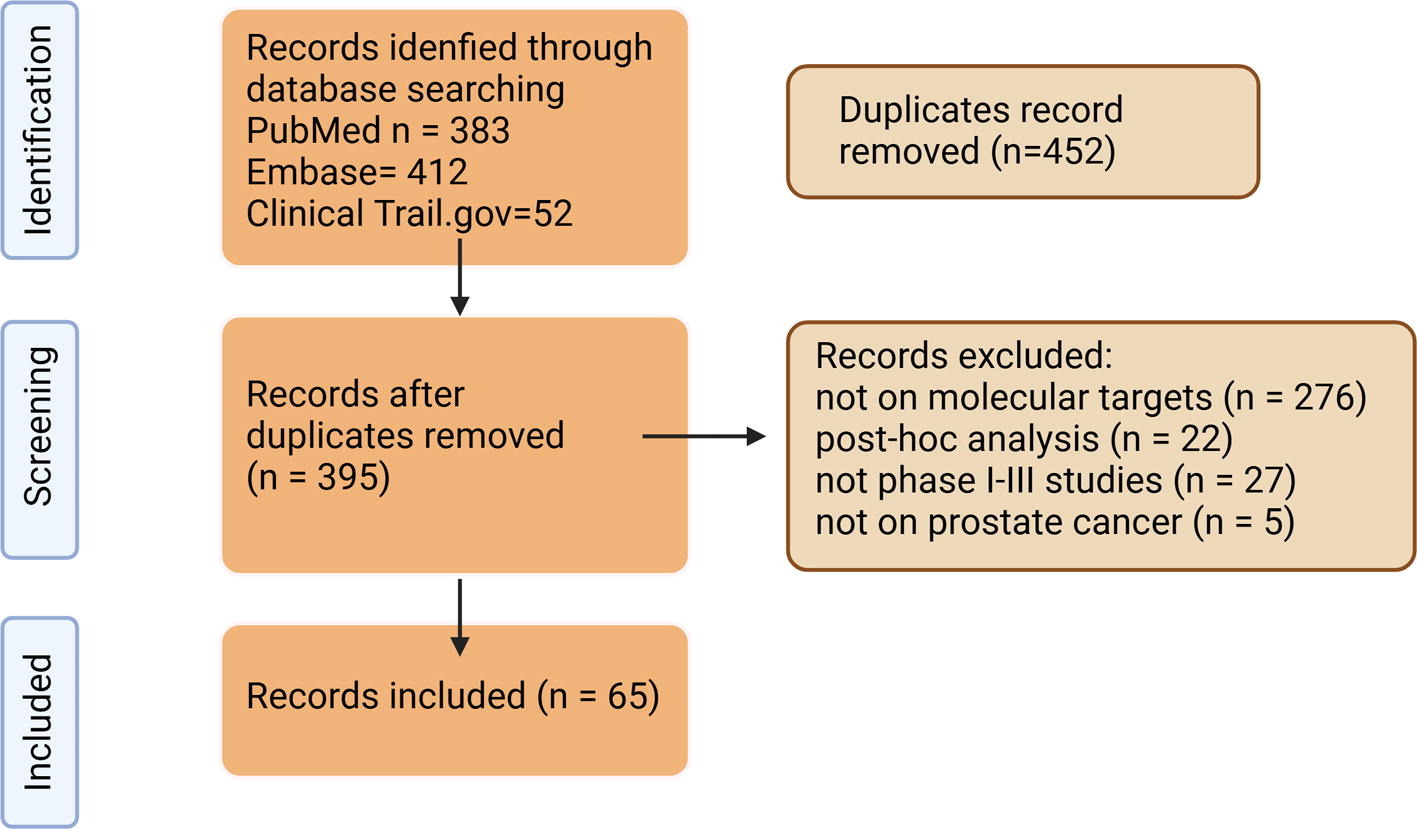

Supplement: Supplementary Figure [file NIHMS2144922-supplement-Supplementary_Figure.jpg]
